# How best to study the function of consciousness?

**DOI:** 10.3389/fpsyg.2015.00604

**Published:** 2015-05-07

**Authors:** Jason Samaha

**Affiliations:** Department of Psychology, University of Wisconsin-MadisonMadison, WI, USA

**Keywords:** consciousness, working memory, metacognition, unconscious processing, relative blindsight

A central project within the scientific study of consciousness is that of uncovering the role that consciousness has in behavior. Under some accounts, the temporary retention of information (short-term or working memory) is accomplished via the maintenance of a conscious representation of that information, and is a candidate function of consciousness (Atkinson and Shiffrin, 1968; Baars and Franklin, 2003). However, recent experiments demonstrating that stimuli rated by observers as invisible can nevertheless be retained over a delay period, suggest that working memory (WM) may, under some conditions, operate unconsciously (Soto et al., 2011; Bergström and Eriksson, 2014). Accepting this conclusion would raise important questions about the classical view that WM may be a biological function of consciousness. Complicating this line of research, however, are long-standing concerns in the study of unconscious cognition as to whether one can be certain in establishing complete unawareness. Here, I discuss how recent conceptual and methodological advances in the study of visual awareness might profitably be adapted to study the role of awareness in WM and to study the functional consequences of conscious perception more generally.

The strength of the claim that a cognitive process occurs in the absence of awareness rests on the extent to which absolute unawareness was actually achieved. A problematic issue for any study investigating unconscious processing is that of assuring that the reporting methodology employed (1) adequately captures all relevant consciously perceived information, and (2) that observers truly have complete unawareness when they are claiming so (Overgaard et al., 2006). If these conditions are not met, behavior seeming to occur unconsciously may actually be the result of degraded, but conscious perceptual information processing. These issues are particularly salient with regard to recent experiments testing for unconscious working memory (e.g., Soto et al., 2011). In these experiments, the researcher's arguments rely on observers being completely unaware of all relevant features of the remembered stimulus (otherwise performance could be attributed to residual conscious processing). As there is currently no consensus regarding the most exhaustive measure of consciousness (Seth et al., 2008), proving complete unawareness of all relevant features of a stimulus may be a suboptimal approach to studying the function of consciousness.

An alternative approach, coined “relative blindsight” (Lau and Passingham, 2006), comes from experimental paradigms in which observers are presented with two or more stimulus conditions for which their perceptual discrimination performance (e.g., d-prime) is matched, yet, subjectively, their confidence or reports of having seen the stimulus vary (Lau and Passingham, 2006; Zylberberg et al., 2012). In these paradigms, awareness is not entirely obliterated, but rather a relative difference exists between two otherwise performance-matched conditions. This contrast in the level of awareness effectively isolates the subjective construct of having seen a stimulus while ruling out confounds due to performance factors like attention, arousal, and motivation, which would presumably also affect objective performance. Furthermore, whereas many existing approaches render stimuli unconscious by degrading them until objective performance is at floor, this approach preserves some performance and thereby alleviates uncertainty that a null finding is simply due to weak stimuli. These paradigms can be applied to the study of the function of subjective awareness in the following way: if, under conditions of relative blindsight, one condition has fewer trials during which a stimulus is subjectively seen, any post-perceptual cognitive process hypothesized to rely on subjective awareness should show a corresponding decline in functionality, otherwise the cognitive process cannot be said to depend on subjective awareness. Although reports about visual awareness are not perfect reflections of phenomenology, and may be better characterized as metacognitive, the subjective feeling of knowing that one has perceived a stimulus often accompanies our conscious sensations, and should be considered a central aspect of visual consciousness.

Methodologically, relative blindsight has been induced by manipulating the absolute amount and the ratio of target to non-target sensory evidence (Zylberberg et al., 2012), or by contrasting different stimulus onset asynchronies (SOA) during metacontrast masking (Lau and Passingham, 2006) (but see Jannati and Di Lollo, 2012). Using these stimulus conditions as the memoranda in WM tasks provides a means of directly testing whether subjective visual awareness improves visual WM. If subjectively experiencing a visual stimulus enhances the retention of that information, then memory performance should be better for the high-awareness, as contrasted with the low-awareness conditions. Because initial perceptual discrimination has been equated, any performance differences related to awareness can be isolated to post-perceptual WM processes such as memory maintenance.

This paradigm can be adapted to address further questions as to whether awareness confers any other type of benefit to WM. By introducing distractors to the delay period of a task, or by having participants hold multiple stimuli in mind, one can test the role that awareness might play in the capacity and distractor resilience of WM processes. Additionally, one could require responses on a continuous scale as a test of the hypothesis that subjective awareness enhances the fidelity of WM. Another intriguing avenue for exploration would be to present multiple items during encoding and employ a retrocue design (Oberauer, 2002) to assess whether the ability to shift attention between items in WM depends on the items being represented consciously. If attention could be allocated to unconscious items in WM (as indexed by enhanced memory of attended information), this would shed light on another important and ongoing debate regarding the dissociation between attention and awareness, but in the WM domain. In this way, relative blindsight paradigms can be adapted to many tasks investigating the role of subjective visual awareness in cognitive processes while circumventing confounds and assumptions associated with creating conditions of complete unawareness.

A limitation of the relative blindsight approach is that by virtue of requiring a contrast between two performance-matched stimulus conditions, physical stimulus properties (such as SOAs or signal-to-noise ratios) also vary across conditions. With respect to metacontrast masking, for example, it has been argued that relative blindsight arises from a confound in the physical stimulus attributes that drive responses at short versus long SOAs (Jannati and Di Lollo, 2012). For this reason, a more recent method of inducing relative blindsight, based on manipultating signal-to-noise ratio (Zylberberg et al., 2012), may be preferred. This issue is also problematic for studies investigating the neural correlates of consciousness, especially those focused on brain areas whose activity is known to reflect physical stimulus differences, such as early visual cortex. By the same logic, this issue may be less problematic when investigating regions whose activity can be shown to be insensitive to physical stimulus properties. This concern can also be mitigated analytically by testing whether the cognitive function or neural activity under investigation also varies with awareness within a single stimulus configuration.

Another nuance in need of discussion is how awareness is assessed within a relative blindsight paradigm. Researchers can compare conditions in which stimuli are more or less often rated as unconscious (as has been done before using subjective “guessing” rates as an measure of percent “seen”; Lau and Passingham, 2006), or they can induce a relative reduction in visibility ratings using some scale, though the stimuli may always, in some sense, be conscious. Likely both approaches are appropriate and can address different issues. The former procedure may be better suited to detect cognitive functions that *fully* depend on conscious processing, while the latter procedure may only show that consciousness has *some* influence on the cognitive process under investigation. The latter procedure may be more sensitive, however, in that it does not require degrading stimuli to the point where they are sometimes rated as unconscious, which could optimize the probability of the “less visible” stimulus engaging other downstream cognitive functions.

Lastly, the choice of measurement for both subjective and objective processing deserves careful consideration. The extent to which both measures are maximally sensitive, for example, could alter experimental results. Popular scales of subjective awareness are often pseudo-continuous (e.g., PAS; Overgaard et al., 2006), yet discrimination performance is often binary (e.g., 2AFC). If some parameter of behavioral performance did in fact vary with awareness but was not captured by the objective measure, relative blindsight would not have been truly established. By having participants reproduce the exact angle of a target Gabor patch, for example, one could compare a continuous parameter of perceptual performance (distance from the correct angle) under levels of continuously varying subjective visibility. As work with this novel paradigm continues to develop, the relative blindsight approach is best seen as a flexible tool that can complement existing methods of investigating the role that consciousness has in behavior while ruling out certain performance confounds and insensitivity due to extreme stimulus degradation.

## Conflict of interest statement

The author declares that the research was conducted in the absence of any commercial or financial relationships that could be construed as a potential conflict of interest.

## References

[B1] Atkinson R. C. Shiffrin R. M. (1968). Human memory: a proposed system and its control processes. Psychol. Learn. Motiv. 2, 89–195 10.1016/S0079-7421(08)60422-3

[B2] Baars B. J. Franklin S. (2003). How conscious experience and working memory interact. Trends Cogn. Sci. 7, 166–172. 10.1016/S1364-6613(03)00056-1 12691765

[B3] Bergström F. Eriksson J. (2014). Maintenance of non-consciously presented information engages the prefrontal cortex. Front. Hum. Neurosci. 8:938. 10.3389/fnhum.2014.00938 25484862 PMC4240068

[B4] Jannati A. Di Lollo V. (2012). Relative blindsight arises from a criterion confound in metacontrast masking: implications for theories of consciousness. Conscious. Cogn. 21, 307–314. 10.1016/j.concog.2011.10.003 22051554

[B5] Lau H. C. Passingham R. E. (2006). Relative blindsight in normal observers and the neural correlate of visual consciousness. Proc. Natl. Acad. Sci. U.S.A. 103, 18763–18768. 10.1073/pnas.0607716103 17124173 PMC1693736

[B6] Oberauer K. (2002). Access to information in working memory: exploring the focus of attention. J. Exp. Psychol. 28, 411–421. 10.1037/0278-7393.28.3.411 12018494

[B7] Overgaard M. Rote J. Mouridsen K. Ramsøy T. Z. (2006). Is conscious perception gradual or dichotomous? A comparison of report methodologies during a visual task. Conscious. Cogn. 15, 700–708. 10.1016/j.concog.2006.04.002 16725347

[B8] Seth A. K. Dienes Z. Cleeremans A. Overgaard M. Pessoa L. (2008). Measuring consciousness: relating behavioural and neurophysiological approaches. Trends Cogn. Sci. 12, 314–321. 10.1016/j.tics.2008.04.008 18606562 PMC2767381

[B9] Soto D. Mäntylä T. Silvanto J. (2011). Working memory without consciousness. Curr. Biol. 21, 912–913. 10.1016/j.cub.2011.09.049 22115455

[B10] Zylberberg A. Barttfeld P. Sigman M. (2012). The construction of confidence in a perceptual decision. Front. Integr. Neurosci. 6:79. 10.3389/fnint.2012.00079 23049504 PMC3448113

